# Gene expression profiles of dicyemid life-cycle stages may explain how dispersing larvae locate new hosts

**DOI:** 10.1186/s40851-019-0146-y

**Published:** 2019-11-13

**Authors:** Tsai-Ming Lu, Hidetaka Furuya, Noriyuki Satoh

**Affiliations:** 1 Marine Genomics Unit, Okinawa Institute of Science and Technology Graduate University, Onna, Okinawa, 904-0495 Japan; 2 Present address: Sars International Centre for Marine Molecular Biology, University of Bergen, 5008 Bergen, Norway; 3 Department of Biology, Graduate School of Science, Osaka University, Toyonaka, Osaka, 560-0043 Japan

**Keywords:** *Dicyema japonicum*, Parasite in octopus renal sacs, Asexual and sexual reproduction, Two adult and larval forms, RNA-seq analyses, Differential gene expression

## Abstract

Metazoans have evolved a great variety of life histories in response to environmental conditions. A unique example is encountered in dicyemid mesozoans. In addition to a highly simplified adult body comprising only ~ 30 cells, dicyemids exhibit a parasitic lifestyle that includes nematogens (asexual reproductive adults), rhombogens (sexual reproductive adults), vermiform larvae generated by nematogens, and infusoriform larvae generated by rhombogens. However, due to the difficulties of observing microscopic endoparasites, the complex life cycle and biological functions of life-cycle stages of dicyemids have remained mysterious. Taking advantage of the recently decoded genome of *Dicyema japonicum*, we examined genes that undergird this lifestyle. Using stage-specific gene expression profiles, we found that biological processes associated with molecular transport, developmental regulation, and sensory response are specified at different stages. Together with the expression of potential neurotransmitters, we further suggest that apical cells in infusoriform larva probably serve sensory functions, although dicyemids have no nervous system. Gene expression profiles show that more genes are expressed in free-living infusoriform larvae than in the other three stages, and that some of these genes are likely involved in locating new hosts. These data provide molecular information about the unique lifestyle of dicyemids and illustrate how an extremely simplified endoparasite adapted and retained gene sets and morphological characters to complete its life cycle.

**Supplementary information:**

**Supplementary information** accompanies this paper at 10.1186/s40851-019-0146-y.

## Background

Due to the simplicity of their body plans, dicyemids and orthonectids were previously called “mesozoans,” a group of intermediate complexity between unicellular protozoans and multicellular metazoans [1–3]. Although the Dicyemida and Orthonectida have recently been classified as two independent phyla [4], the phylogenetic position of these enigmatic groups remained controversial for a long time [5, 6], due to the extremely high rates of molecular changes among mesozoans [7–10]. The possession of a “spiralian peptide” by dicyemids suggests that dicyemids are morphologically simplified spiralians [11]. Phylogenomic studies by our group showed that the clade of dicyemids and orthonectids has affinity for the Rouphozoa (Platyhelminths and Gastrotricha), rather than for mollusks and annelids [12], while other studies concluded that orthonectids are highly derived members of the phylum Annelida, and not closely related to dicyemids [13, 14].

**Fig. 1:**
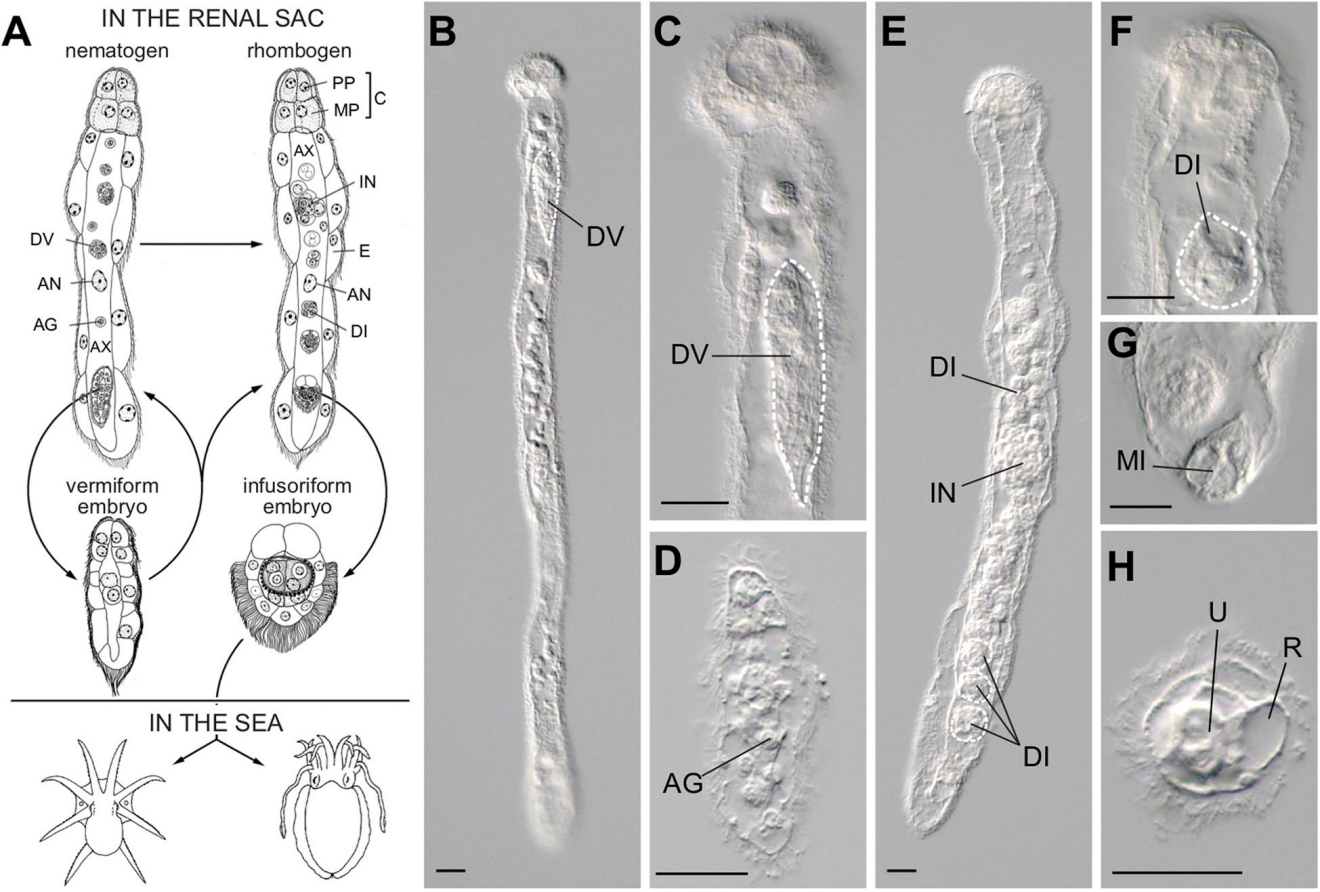
The life cycle of dicyemid mesozoans. **a** Life cycle of dicyemids, see the text for details. Adapted and modified from Furuya and Tsuneki (2003). **b-h** Adult and larvae of *Dicyema japonicum*. **b** A nematogen, the asexual reproductive adult consisting of 22 somatic cells. **c** A vermiform embryo develops inside a nematogen. **d** A vermiform larva. **e** A rhombogen, the sexual reproductive adult. **f** An infusoriform embryo develops inside a rhombogen. **g** An infusoriform larva dispersing from a rhombogen. **h** A mature, free-living infusoriform larva escaped from the parent rhombogen. AG, agamete; AN, axial cell nucleus; AX, axial cell; C, calotte; DI, developing infusoriform embryo; DV, developing vermiform embryo; E, epidermal cell; IN, infusorigen; MI, mature infusoriform larva; MP, metapolar cell; PP, propolar cell. R, refringent body; U, urn cell. Scale bars: 20 μm

**Fig. 2:**
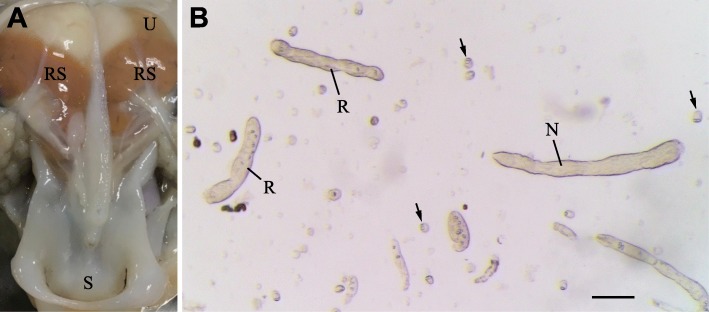
Dicyemids are microscopic endoparasites inhabiting the renal sacs of octopuses. **a** Dissecting the renal system of an octopus. The renal sacs are full of urine, and dicyemids inhabit the renal appendages (orange tissue) inside the renal sacs. **b** Urine of an octopus drawn from the renal sac. Dicyemids at different life-cycle stages appear in the urine; therefore, samples of different life cycle stages had to be carefully isolated and sorted. N, nematogen; R, rhombogen; RS, renal sac; S, siphon; U, urine. Arrows, infusoriform larvae. Scale bars: 50 μm

Dicyemids are microscopic endoparasites that inhabit the renal sacs of cephalopods (Figs. 1 and 2). Although more than one dicyemid species can inhabit an individual host [15], dicyemids tend to be highly host-specific [16]. Adult dicyemid bodies comprise only ~ 30 cells (Figs. 1 and 2), and consist of three regions: the collate region, a central axial cell, and ciliated epidermal cells (Fig. 1a). The collate region (the most anterior eight cells) is used to attach to the surface of cephalopod renal tissues. The central axial cell is surrounded by an outer layer of ciliated epidermal cells, and functions mainly in reproduction, producing vermiform or infusoriform embryos. Ciliated epidermal cells absorb nutrients directly from host urine by endocytosis and cilia appear to generate nutrient-containing water flow over the dicyemids [3, 17]. Dicyemids lack a digestive tract, coelom, circulatory system, and other differentiated tissues (Fig. 1). This is probably the most extreme case of secondary reduction of body plan complexity in a parasitic spiralian.

In addition to this simple body plan, the life cycle of dicyemids is characterized by two reproductive modes (asexual and sexual) and there are larval and adult stages in each reproductive process (Fig. 1a). After infecting a cephalopod host, the germinal cell develops into an asexual reproductive adult nematogen (Fig. 1a, b). The agamete (axoblast) subsequently develops into asexual reproductive vermiform embryos, inside the central axial cell of the nematogen [18] (Fig. 1a, c, d). As the embryos mature, the vermiform larvae escape from the axial cell of the parent nematogen and develop into new nematogens that attach to renal tissue of the same host, which increases the population density. Once the population density inside a host reaches a certain threshold, nematogens transform into sexually reproductive adults, called rhombogens [2] (Fig. 1a, e). Inside the central axial cell of a rhombogen, the hermaphroditic gonad (infusorigen) generates sperm and eggs. Fertilized gametes develop into infusoriform larvae [19] (Fig. 1f–h). Mature, free-swimming infusoriform larvae need to be able to detect and locate new hosts in the open sea (Fig. 1a, h).

However, due to practical difficulties of tracing dispersing larvae in the ocean, how infusoriform larvae search for and infect new hosts to sustain the survival of dicyemid lineages remains unclear. A small patch of short cilia, called “bristles”, located dorsal to the nuclei of apical cells of infusoriform larvae, are described in some dicyemid species [20, 21]. They are distinctly different from other types of cilia on other peripheral cells of infusoriform larvae and cilia on cells of adult collate and trunk regions. Due to their short length, bristles are unlikely to enable mobility, and their function remains ambiguous [20]. Since cilia are also considered signal transduction components and participate in mechanoreception [22], bristles may participate in host detection. Although no nervous system has been reported in dicyemids and their cell surface membrane receptors, such as G-protein-coupled receptors (GPCR), have undergone massive reduction [14], dicyemid behavior suggests that they utilize some unknown sensory machinery to receive and respond to signals from the surrounding environment.

Development of new experimental techniques, such as next-generation sequencing, provides additional means of interpreting dicyemid life cycles from diverse perspectives. Recently, we sequenced the draft genome of *Dicyema japonicum* [23]. This dicyemid genome is highly reduced to approximately 67.5 Mb with 5012 protein-coding genes. Although various biological pathways have been retained, as in non-parasitic spiralians, the number of genes in each pathway is highly reduced. The present study characterized gene expression profiles of the four life-cycle stages of *D. japonicum*. Moreover, given the dramatically different gene expression profiles in these stages, we further examined immunohistochemically the localization of neurotransmitters, particularly in infusoriform larva. These results provide insights into biological functions of dicyemid stages and potential sensory functions for detecting new hosts.

## Materials and methods

### Biological materials

*Dicyema japonicum* specimens of all four life-cycle stages, nematogens (asexual reproductive adults), rhombogens (sexual reproductive adults), vermiform larvae generated by nematogens, and infusoriform larvae generated by rhombogens (Fig. 1a–h; Fig. 2), were collected from renal sacs of adult *Octopus sinensis* (Fig. 2). In order to extract sufficient RNA for library preparation and to eliminate biological variability among animal individuals, we combined dicyemids obtained from seven *O. sinensis* specimens. Each individual *Dicyema* was manually sampled using a glass pipet under a stereomicroscope and identified to life-cycle stage (Fig. 1a–h). Specimens of each life-cycle stage were then homogenized in TRIzol Reagent (Ambion, #15596026) and stored at − 80 °C.

### Transcriptome sequencing, assembly, and annotation

RNA was extracted from specimens using a Direct-zol RNA MicroPrep Kit (Zymo Research, #R2060). The same amount of RNA (1.3 ng) extracted from a pool of dicyemids from seven host octopuses for each of the four life-cycle stages was used for library preparation. After reverse transcribing RNA to cDNA with a SMART-Seq v4 Ultra Low Input RNA Kit (Clontech Laboratories, #634888), a Nextera XT DNA Library Preparation Kit (Illumina, #FC-131-1024) was utilized for library preparation. Sequencing was performed on a single HiSeq 4000 run to avoid technical bias (Table 1).

Raw reads were quality filtered (Q score ≥ 20) and trimmed with Trimmomatic (v0.33). De novo transcriptome assembly was performed using Trinity (v2.0.6) [24] with default settings. TransDecoder was utilized to extract coding regions and to translate transcripts into amino acid sequences [25]. To avoid contamination of dicyemid samples with octopus cells, we washed the samples with filtered seawater several times, but we still could not preclude minimal contamination. Therefore, we mapped genomic sequencing reads of host octopus back to the dicyemid transcriptome assembly using Bowtie 2 (v2.2.3) [26]. Only 1% of the dicyemid transcripts mapped to octopus reads; these were removed from the data.

Transcript abundances of each life-cycle stage were assessed with kallisto [27], and reported as TPM (Transcripts Per Kilobase Million) measures. Further, TPM values of all stages were normalized to TMM (trimmed mean of M-values) measures. Differentially expressed transcripts were extracted and partitioned into clusters according to the expression patterns of four life-cycle stages using Perl scripts in the Trinity package, analyze_diff_expr.pl and define_clusters_by_cutting_tree.pl, respectively. Clustered matrices and heat maps were created using R (v3.2.4) with the package Bioconductor (v3.0) and pheatmap (v1.0.8). Gene ontology (GO) over-representation analyses were conducted using DAVID [28] and PANTHER [29].

### Immunostaining and imaging

Antibodies against the following were used in the present study: acetylated tubulin (Sigma, #T6793, 1:1000 diluted in blocking solution), oxytocin (Immunostar #20068, 1:4000 dilution), vasopressin (Immunostar #20069, 1:2000 dilution), dopamine (Abcam #ab8888, 1:1000 dilution), dopamine-beta-hydroxylase (Immunostar #22806, 1:2000 dilution), and gamma-aminobutyric acid (GABA) (Sigma, #A2052, 1:1000 dilution). Specimens were fixed in 4% paraformaldehyde (PFA) for 30 min and then stored in 75% ethanol at − 20 °C. For immunostaining, they were incubated first in blocking solution (3% BSA and 0.1% Triton X-100 in PBS, 1 h), then in primary antibody solution, and finally in acetylated tubulin mouse monoclonal antibody at 4 °C overnight. Fluorescent signals were detected after incubation in a secondary antibody solution of Alexa Fluor 594-conjugated, goat anti-mouse antibody. DAPI (Invitrogen, 1 μg/mL in PBST) was used for nuclear staining, and plasma membranes were stained with CellMask (Life technology, C10046). Fluorescent images were acquired using a Zeiss 780 confocal microscope with 20X and 100X objectives.

## Results

### Differential gene expression profiles in the four stages

In order to compare the gene expression profiles of dicyemid life-cycle stages, we sequenced transcriptomes of respective adult and larva stages of asexual and sexual reproductive forms. Quality-trimmed RNA-seq reads of four life-cycle stages were pooled and assembled de novo as a reference transcriptome assembly. We then separately aligned reads of each life-cycle stage against the reference transcriptome assembly to estimate the expression level of each gene. Notably, since multiple developing embryos exist in axial cells of adults, the abundance of gene expression at adult stages (nematogen and rhombogen) also reflects some gene expression from developing embryos.

**Fig. 3:**
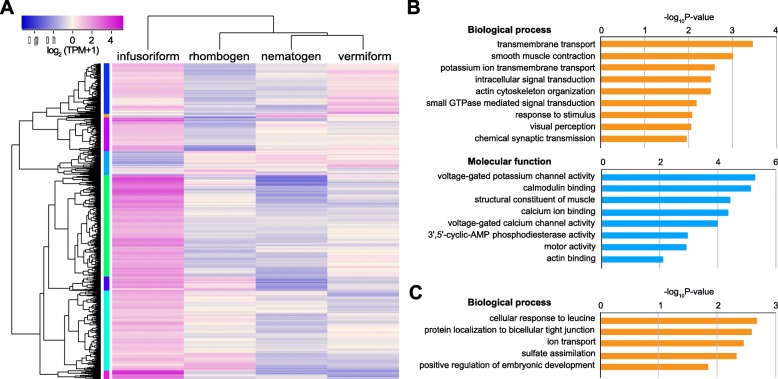
Differential gene expression and over-represented GO terms in four life-cycle stages of *Dicyema japonicum*. **a** A heat map of expression levels of differentially expressed genes manifesting ≥4x differences among life-cycle stages. More than 80% of differentially expressed genes exhibit higher expression in infusoriform larvae. **b** Highly expressed infusoriform larval genes that exhibit over-representation for GO terms such as response to stimulus, chemical synaptic transmission, and ion channel activity, suggesting that dicyemids possess potential sensory functions. GO terms associated with mobility, such as motor activity and actin binding, suggest that some cilia of infusoriform larvae contribute to their swimming ability. **c** The over-represented GO terms of highly expressed genes in life cycle stages other than infusoriform larvae

In comparing gene expression among the four stages, we identified 1641 differentially expressed genes manifesting ≥4× changes among stages. Most of them (81.6%) exhibited higher expression during the infusoriform (free-swimming) larval stage (Fig. 3a). Gene expression profiles were also congruent with stages. That is, the expression profile of vermiform larvae was more correlated with that of nematogens, which produce vermiform larvae than with that of rhombogens, which produce infusoriform larvae (Additional file 1: Figure S1). The infusoriform larval stage was the least correlated with all others.

To speculate about potential biological functions of different life-cycle stages, we summarized the GO over-representation results from DAVID and PANTHER analyses. Up-regulated genes in infusoriform larvae over-represent many GO terms associated with sensory function, such as response to stimulus, synaptic transmission, signal transduction, and ion channel activities (Fig. 3b). In addition, GO terms associated with mobility, such as motility activity and actin binding, are also over-represented in infusoriform larvae (Fig. 3b). On the other hand, although GO over-representation analyses for differentially expressed genes in the other three life-cycle stages obtained weaker statistical support than that of the infusoriform larval stage, the highly expressed genes in these stages are likely associated with molecular transport and developmental processes (Fig. 3c).

### Localization of potential neurotransmitters and neuropeptides

**Fig. 4:**
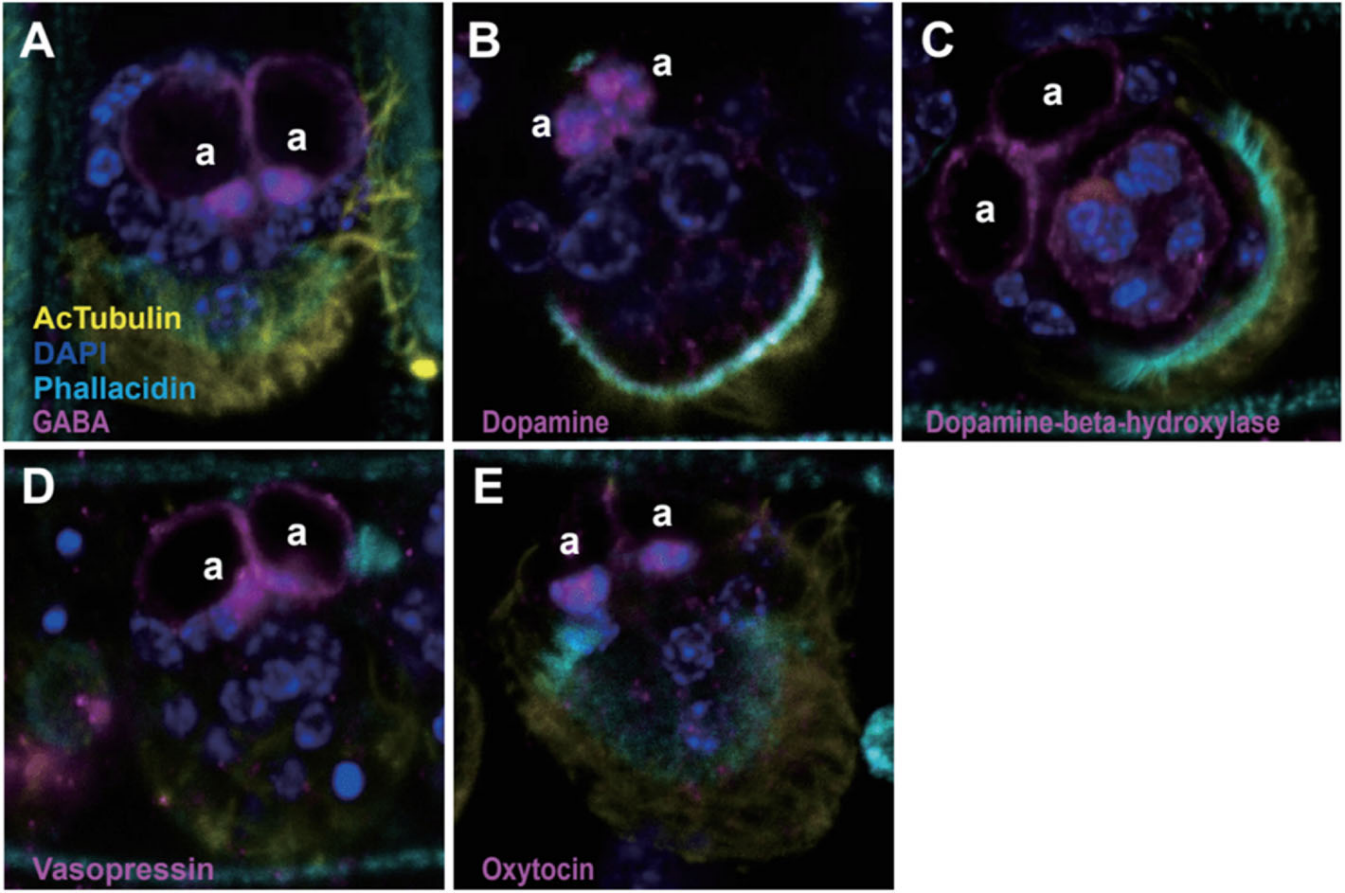
Colocalization of potential neurotransmitters and neuropeptides in apical cells of infusoriform larva. Immunostaining signals of potential neurotransmitters and neuropeptides appear in apical cells of infusoriform larva, GABA (**a**), dopamine (**b**), dopamine beta hydroxylase (**c**), vasopressin (**d**), and oxytocin (**e**). Dopamine beta hydroxylase is also expressed in urn cells (**c**) inside the infusoriform larva. These results suggest that apical cells are likely the signal transduction center to receive environmental signals and to coordinate the release of germinal cells. Nuclei were labeled with DAPI (blue), and acetylated tubulin antibodies were used to label cilia (yellow). a, apical cell

As differentially expressed genes are over-represented in GO terms related to sensory function, especially in the infusoriform larval stage, we immunohistochemically assayed for the presence of neurotransmitters and neuropeptides, which are potentially involved in sensory functions. Since structures of neurotransmitters and neuropeptides are often conserved among metazoans, we used commercial polyclonal antibodies. Immunostaining signals of dopamine, gamma-aminobutyric acid (GABA), dopamine beta hydroxylase (DBH), vasopressin, and oxytocin are mainly observed on apical cells of infusoriform larvae (Fig. 4). In addition to apical cells, DBH was also expressed on urn cells, which are the carriers of germinal cells that are released from infusoriform larvae to infect new hosts (Fig. 4c). Using the same settings for image acquisition on the confocal microscope, it was difficult to obtain definitive immunostaining signals of these neurotransmitters and neuropeptides at samples of the other three life-cycle stages.

### Bristles on the apical cells of *D. japonicum*

**Fig. 5:**
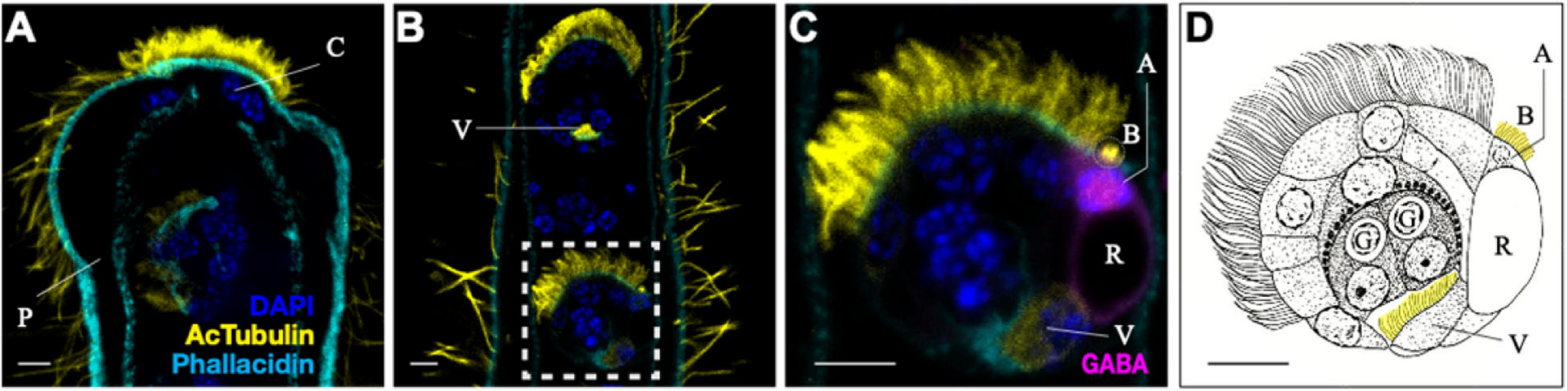
Various types of cilia and existence of bristles on *Dicyema japonicum*. **a** In the anterior end of a rhombogen, cilia on the calotte cells are shorter and denser than those on other peripheral epithelial cells. **b** Ventral internal cells of infusoriform larva bear short cilia forward to urn cavity. A dashed square encloses an infusoriform embryo inside the central axial cell of a rhombogen adult. The density of cilia on epithelial cells of the infusoriform larva is higher than on epithelial cells of the rhombogen adult, reflecting higher mobility of larvae. **c** An infusoriform embryo, enlargement of the dashed square of (**b**). The length of bristles on apical cells of the infusoriform larva is approximately one-third that of cilia on epithelial cells. Immunostaining signals of GABA appear in apical cells. **d** Sagittal optical section of an infusoriform larva. Yellow labels bristles and cilia on ventral internal cells, respectively. Adapted and modified from Furuya et al. (1992). A, apical cell; B, bristles; C, calotte cell; G, germinal cell; P, peripheral epithelial cell; R, refringent body inside apical cell; V, ventral internal cell of infusoriform larvae. Scale bars: 20 μm

We applied acetylated tubulin antibodies to counterstain cilia. This indicated that dicyemids bear various types of cilia on different regions of their bodies (Fig. 5). Cilia on the collate regions of adults are short and denser, while cilia on the trunk region are longer (Fig. 5a). On infusoriform larvae, peripheral somatic cells bear long cilia, which are utilized for movement in the ocean. Cilia on the ventral internal cells are shorter and possibly circulate fluid within the urn cavity, providing nutrients and oxygen for both urn cells and germinal cells [30] (Fig. 5b, d). Notably, we found that short cilia, or bristles, which the original description of *D. japonicum* said they were not visible, actually do exist on the apical cells of infusoriform larvae, and the length of bristles is approximately one-third that of cilia on peripheral epithelial cells (Fig. 5c, d).

## Discussion

Due to the difficulties of tracking and observing dicyemids in the ocean, their complex life cycle remains unknown, and how new commensalism becomes established remains undiscovered. We assumed that stage-specific transcriptome analysis might offer insights into the biological functions of the four dicyemid life-cycle stages. Except for infusoriform larvae, which are released to search for new hosts in the open water, the other three stages remain inside the renal system of the host. The present study of differential gene expression profiles showed that infusoriform larvae up-regulate a gene set distinct from those of the other three stages. These genes are over-represented in GO terms related to visual perception, response to stimulus, and chemical synaptic transmission, which suggests that infusoriform larvae utilize specialized sensory functions to detect new hosts and environments. In addition, over-represented GO terms such as signal transduction, transmembrane transport, and motor activity, suggest that infusoriform larvae may be able to process signals and their sources to initiate downstream responses. On the other hand, over-represented GO terms for the other three stages suggest that they perform mainly physiological and reproductive functions.

Although the chemistry of the octopus renal system has been examined, no candidate chemical relevant to the dicyemid transformation from asexual to sexual reproduction has been reported yet [31]. However, dicyemids may sense concentrations of chemical signals related to population density, triggering a shift in reproductive modes [2]. Moreover, *Pax6*, a tool-kit gene playing key roles in development of sensory organs, has been reported in dicyemids [32], which implies that conserved tool-kit genes may allow dicyemids to develop some hitherto undescribed sensory machinery without the existence of sensory organs. Serotonin is a well-known neurotransmitter functioning as a hormone or intracellular regulator, and a serotonin-like molecule has been identified in dicyemids [33]. It is present either in the small vesicles of ciliated epidermal cells of adults, or in the capsule cells and central internal cells of infusoriform larvae.

In addition to serotonin, this study reveals that expression of five other neurotransmitters and neuropeptides is co-localized on apical cells of infusoriform larvae, although their regulation and interactions in dicyemids are still unknown. Dopamine may excite infusoriform larvae to execute swimming, which is essential to leave the parent to seek new hosts. GABA is an inhibitory neurotransmitter that opposes dopaminergic activity [34]. GABA is known to affect swimming behavior in paramecia, which also lack nervous systems [35]. DBH could modulate dopamine levels by converting dopamine to norepinephrine. In addition to apical cells, DBH is also expressed in urn cells of infusoriform larvae, suggesting that DBH may generate norepinephrine for downstream regulation when urn cells receive secreted dopamine from neighboring apical cells. Since invertebrates, other than cephalopods, have only a single oxytocin/vasopressin (OT/VP) superfamily peptide homolog [36], the immunostaining signals of vasopressin and oxytocin antibodies, which both appeared on apical cells (Fig. 4d, e), should recognize the same OT/VP peptide in dicyemids. As in other animals, the OT/VP peptide may be associated with reproduction in infusoriform larvae. If the release of germinal cells employs similar hormonal control to birthing, the OT/VP peptide may mediate release of dopamine, as reported in vertebrates. Undoubtedly, further examination of possible regulation and interactions of these neurotransmitters and neuropeptides would help to explain signal transduction of sensory functions in dicyemids.

Cilia on different cells of dicyemids may perform different functions due to differences in their morphology (Fig. 5). Bristles on apical cells probably do not contribute to mobility (Fig. 5c, d), as they are too short to generate water currents. Cilia are also considered signal transduction components and participate in mechanoreception [22]. Together with co-localization of potential neurotransmitters and neuropeptides on apical cells, we think that apical cells may act in signal processing and the sensing of new hosts. Inferring from the present results and previous studies, we propose a hypothesis to explain how dicyemids approach new hosts and release germinal cells to establish new commensal individuals. When infusoriform larvae are released into open water, the gravity of refringent bodies inside apical cells may direct these larvae to the seabed [1], where they usually swim by spinning clockwise. Employing a reduced number of GPCR receptors [14] and regulatory machinery involving the neurotransmitters described above, apical cells sense new hosts and transduce signals to trigger release of germinal cells. Within the capsule cells, abundant eosinophilic granules with lytic enzymes may release germinal cells [37]. Cilia on ventral internal cells circulating the fluid within the urn cavity may not only bring nutrients and oxygen to the germinal cells [30], but may also facilitate release of germinal cells to infect new hosts (Fig. 5c, d). Yet, how germinal cells migrate to the renal sacs of octopuses and develop into nematogen adults remains to be discovered, and the cellular and molecular mechanisms involved in this process should be explored in future studies.

Parasitism has been reported in 15 of the generally recognized 35 animal phyla [38]. It is likely to have evolved independently more than 200 times, and each parasitism event reflects the interaction of a given host–parasite pair. Adaptations to a parasitic lifestyle vary case by case. Although parasitism often exhibits convergence in terms of simplified morphology and complex life cycles reflect selective pressures common to parasitism, this decoded dicyemid genome [23] and the expression profiles of four life-cycle stages presented here provide rich resources to examine zoological puzzles during evolution of these enigmatic spiralians. This study shows that, in dicyemids, in which sensory organs have become secondarily reduced commensurate with adaptation to a parasitic lifestyle, dispersing larvae employ ancestral receptor genes and signal transduction systems to search for new cephalopod hosts to complete their life cycles. Further studies may disclose molecular mechanisms underlying such systems.

## Conclusion

The present study presents gene expression profiles for the different life-cycle stages of dicyemids and suggests that dicyemid dispersal larvae utilize sensory machinery to search for new hosts to complete their life cycles. As demonstrated by distinctive gene expression profiles, the role of infusoriform larvae is mainly to search for new hosts in the open ocean, unlike the other three life-cycle stages, which are dedicated to feeding and reproduction inside the hosts. Infusoriform larvae also may mediate the release of germinal cells to establish new infections using neurotransmitters and neuropeptides. In addition, we suggest that apical cells bearing short cilia or “bristles” are likely the key components for sensory function in infusoriform larvae, acting as sensory neurons in other animals.

## Supplementary information

**Additional file 1: Figure S1.** Differentially expressed genes show correlations with life-cycle stages. Spearman’s correlation between four dicyemid life-cycle stages. Gene expression profiles of the three stages inhabiting the renal sac exhibit higher correlations than with dispersing infusoriform larva, indicating that they employ different gene sets to conduct distinct biological functions in different environments. I, infusoriform larva; R, rhombogen; N, nematogen; V, vermiform larva.

## Data Availability

This *Dicyema japonicum* genome project has been registered at NCBI under BioProject accession PRJNA496385. Sequencing reads of the genome and transcriptomes have been deposited in the NCBI Sequence Read Archive (SRR8079359, SRR8079349–52, SRR8079348, SRR8079591, SRR8061618, SRR8061630–3). Genome and transcriptome assemblies have been deposited at DDBJ/ENA/GenBank (RDEW00000000) and NCBI Transcriptome Shotgun Assembly Sequence Database (GHAB00000000), respectively. The genome browser is available at https//oist.marinegenomics.
