# Supplementary material for: Gene expression profiles of dicyemid life-cycle stages may explain how dispersing larvae locate new hosts
Source: Zoological Lett. 2019 Nov 13;5:32. doi: 10.1186/s40851-019-0146-y (PMC6854800; doi:10.1186/s40851-019-0146-y)
Supplement: Supplementary file 1 — Additional file 1: Figure S1. Differentially expressed genes show correlations with life-cycle stages. Spearman’s correlation between four dicyemid life-cycle stages. Gene expression profiles of the three stages inhabiting the renal sac exhibit higher correlations than with dispersing infusoriform larva, indicating that they employ different gene sets to conduct distinct biological functions in different environments. I, infusoriform larva; R, rhombogen; N, nematogen; V, vermiform larva. [file 40851_2019_146_MOESM1_ESM.docx]

**
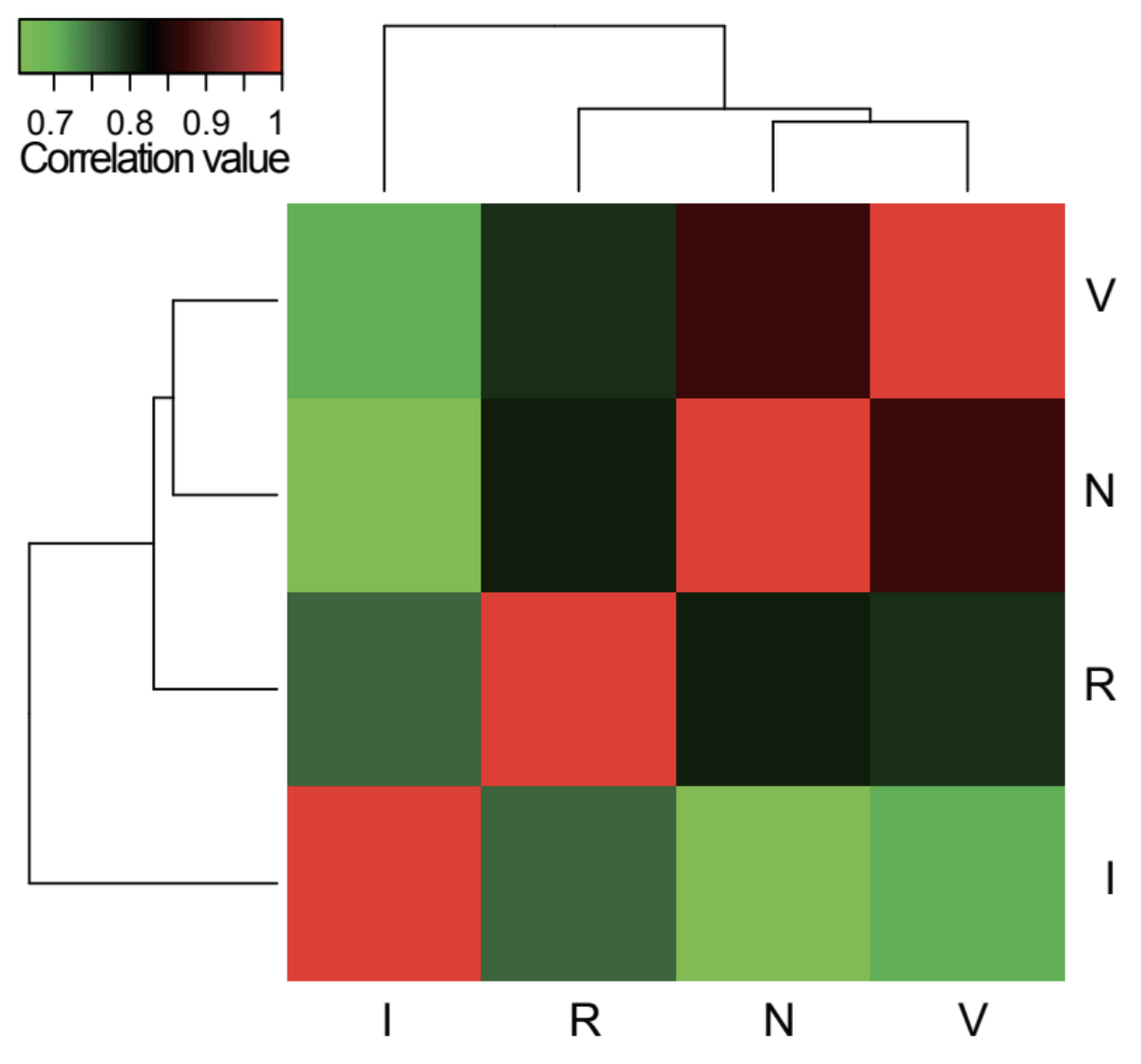
**

**Additional file 1: Figure S1. Differentially expressed genes show correlations with life-cycle stages**
